# New Kendomycin Derivative Isolated from *Streptomyces* sp. Cl 58-27

**DOI:** 10.3390/molecules26226834

**Published:** 2021-11-12

**Authors:** Constanze Paulus, Oleksandr Gromyko, Andriy Luzhetskyy

**Affiliations:** 1 Department of Pharmaceutical Biotechnology, Saarland University, 66123 Saarbrücken, Germany; constanze.paulus@uni-saarland.de; 2 Department of Genetics and Biotechnology, Ivan Franko University of Lviv, 79005 Lviv, Ukraine; oleksandr.gromyko@lnu.edu.ua; 3 AMEG Department, Helmholtz Institute for Pharmaceutical Research Saarland, 66123 Saarbrücken, Germany

**Keywords:** polyketide synthases, *Streptomyces*, ansamycins

## Abstract

In the course of screening new streptomycete strains, the strain *Streptomyces* sp. Cl 58-27 caught our attention due to its interesting secondary metabolite production profile. Here, we report the isolation and characterization of an ansamycin natural product that belongs structurally to the already known kendomycins. The structure of the new kendomycin E was elucidated using NMR spectroscopy, and the corresponding biosynthetic gene cluster was identified by sequencing the genome of *Streptomyces* sp. Cl 58-27 and conducting a detailed analysis of secondary metabolism gene clusters using bioinformatic tools.

## 1. Introduction

**Figure 1:**
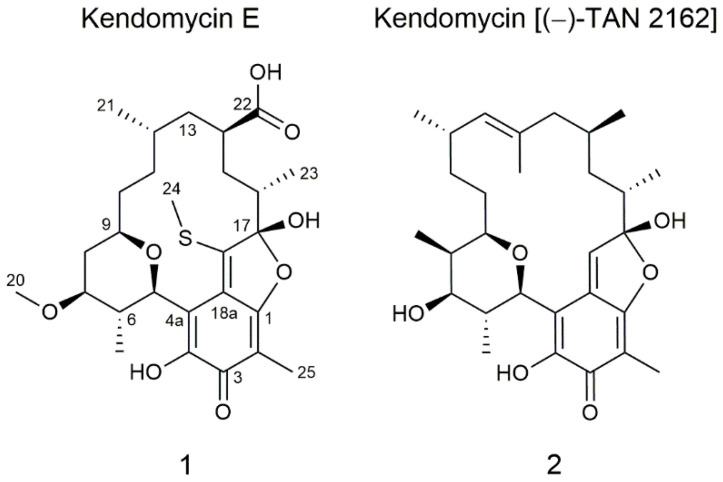
Chemical structures of the new kendomycin E (**1**) and the previously discovered kendomycin [(−)-TAN 2162] (**2**).

Polyketide natural products are a highly diverse group of compounds with a wide range of applications, for example as antibiotics, immunosuppressants or anticancer chemotherapeutics [1,2,3,4]. These highly oxygenated compounds are produced by multimodular enzymes called polyketide synthases (PKSs) that click together starter units such as acetyl-CoA with malonyl-CoA extender units and carry out modifications through domains such as ketoreductases, dehydratases or enoyl reductases, leading to a vast supply of structurally diverse molecules [5]. More specifically, modular type I PKSs, for example, consist of linearly arranged obligatory ketosynthase (KS), acetyl carrier protein (ACP) and acyl transferase (AT) domains, as well as optional ketoreductase (KR), dehydratase (DH), enoyl reductase (ER), methyltransferase (MT) and thioesterase (TE) domains grouped into modules, each performing one elongation and ketoreduction step. Type III PKSs, predominately found in plants, are often called the chalcone/stilbene synthase superfamily and can be divided into five groups according to the post-PKS processing enzyme domains (RppA, PhID, DpgA, ArsB/SarsS, Gcs/ArsC/PKS18) [6]. Sometimes, type I and type III PKS enzymes are combined in one cluster, resulting in partly aromatic and acyclic methylated structures. To date, *Streptomyces* strains have been a major source of polyketide natural products and are still a reliable source of unknown metabolites; hence, the detailed examination of new *Streptomyces* strains is still worthwhile [7]. Screening of *Streptomyces* sp. Cl 58-27 led to the identification of a new derivative (**1**) of kendomycin natural products (Figure 1). Kendomycin [(−)-TAN 2162] (**2**), first described in 1997 in the patent literature and later isolated from *Streptomyces violaceruber* by Bode et al. [8], is a well-known macrocyclic ansamycin natural product with reported activities against osteoporosis, various bacteria including multidrug-resistant *Staphylococcus aureus* (MRSA) and several human cancer cell lines, and was found to be a potent endothelin receptor antagonist [9,10,11,12]. To date, three more derivatives (kendomycins B–D) have been discovered which differ in methylation degree and the presence of a thiomethyl group or N-acetyl-L-cysteine (NAC) attached to the 2,5-dihydrofuran ring [13]. The biosynthetic pathway has been elucidated by Bode and Zeeck [8,9] and Wenzel et al. [14], revealing a joined enzymatic machinery of a type III and a type I PKS. Genome sequencing of Cl 58-27 and analysis of the bioinformatics content using antiSMASH [15] led to the discovery of a cluster similar to the published kendomycin gene cluster. Gene and gene function comparisons enabled us to speculate about the biosynthetic steps necessary for the production of the new kendomycin variant.

## 2. Results and Discussion

### 2.1. Isolation and Structure Elucidation of Kendomycin E

The dereplication of metabolites produced by *Streptomyces* sp. Cl 58-27, isolated from the rhizosphere of *Cedrus libani* (Nicitsky Botanical Garden, Crimea Peninsula, Ukraine), resulted in a putatively new compound that did not match any entry in the Dictionary of Natural Products Database (DNP) [16]. Aiming to isolate this compound, the strain was cultivated in 5 L DNPM medium, and the compound was purified over several chromatographic steps. In the course of structural elucidation using 1D and 2D NMR spectroscopic data including ^1^H-^1^H COSY, HSQC, HMBC and HSQC-TOCSY, it became apparent that the compound has high similarity to the already known kendomycin [(−)-TAN 2162] (Figure 1).

Kendomycin E (**1**) was isolated as a yellow solid with a yield of 1.6 mg/5 L medium. UV maxima were found at 220 and 386 nm, and a molecular ion peak at *m*/*z* 523.2358 [M + H]^+^ (522.228 [M], calc. 522.2287) was determined by high-resolution electrospray ionization mass spectrometry (HRESIMS) (Figures S1 and S2). The molecular formula C_27_H_38_O_8_S, obtained from the exact mass, indicates nine degrees of unsaturation. Analysis of the ^1^H NMR spectrum acquired in methanol-*d_4_* revealed the presence of seven methine protons (δ_H_ 5.41, 3.96, 3.48, 2.81, 1.84, 2.23, 2.09), five methylenes (δ_H_ 2.00–1.34, 1.53–1.01, 1.52–1.41, 1.35, 1.30–1.23) and six methyl groups (δ_H_ 3.40, 2.78, 1.88, 1.25, 0.95, 0.89) (Table S1 and Figure S3). Complementary to this, seven primary, five secondary, six tertiary and eight quaternary carbons were detected in ^13^C and HSQC spectra (Table S1, Figures S4 and S6). The detailed analysis of NMR spectroscopic data revealed first of all the presence of the basic benzofuranone core, typical for kendomycin natural products, here equipped with a methyl group at position C-2 and a hydroxyl group at C-4. This was supported by key HMBC correlations from H-25 to C-1/C-2/C-3/C-18a, H-5 to C-3/C-4/C-4a/C-18a and from H-24 to C-18/C-18a (Figure S7). Furthermore, analysis of ^1^H-^1^H COSY spectrum led to the discovery of a large spinsystem (H-5/H-6 (H-19)/H-7/H-8/H-9/H-10/H-11/H-12(H-21)/H-13/H-14/H-15/H-16(H-23)) connected to the benzofuranone core at positions C-4a and C-17 (Figure S5). Including the corresponding HMBC correlations (e.g., from H-5 to C-4a/C-6/C-9/C-19, from H-6 to C-5/C-19 and from H-7 to C-6/C-8/C-9/C-20), the spinsystem was found to consist on the one hand of a tetrahydropyran ring, methylated at position C-6 and methoxylated at position C-7; and on the other hand of an aliphatic chain, methylated at C-12/C-16 and carboxylated at C-14. This so-called *ansa* feature was found, based on HMBC correlations from H-10 to C-9/C-11/C-12, from H-21 to C-12/C-13, from H-14 to C-13/C-22/C-15 and from H-23 to C-15/C-16/C-17. Comparing the new derivative with the known kendomycin [(−)-TAN 2162] (**2**) and kendomycins B–D, some unique structural features stand out. For instance, minor changes can be seen in the tetrahydropyran ring that lacks a methyl group at C-8 and bears a methoxy group at C-7 instead of a simple hydroxy group. Most importantly, the aliphatic chain lacks two double-bonded, methylated carbons between C-12 and C-13 which was not found like this for any other kendomycin derivative. Concerning the absolute stereochemistry of the new kendomycin derivative, it was assumed that all stereocenters coincide with the stereochemistry previously found in kendomycin [(−)-TAN 2162] (**2**) and kendomycins B–D, which was elucidated by X-ray crystallography and advanced Mosher’s method [8], [13]. It was further assumed that the insertion of the carboxylic group at C-22 does not invert the stereocenter since reaction occurs at the former methyl group as it is described for nocamycin [17].

Kendomycin E was tested against several bacterial, yeast and fungal strains including *Acinetobacter baumannii*, *E. coli* (ΔacrB), *S. aureus* Newman, *E. coli* (wt), *Mycobacterium smegmatis*, *Pseudomonas aeruginosa* PA14, *Bacillus subtilis*, *Citrobacter freundii*, *Mucor hiemalis*, *Candida albicans*, *Cryptococcus neoformans* and *Pichia anomala* (see Section 4), but no activity was observed. This was surprising, since other kendomycin derivatives show good antimicrobial activity. The reason for this must be the structural changes in the ansa chain connecting the ring moieties.

### 2.2. Genome Sequencing of Streptomyces *sp.* Cl 58-27 and Identification of Kendomycin E Gene Cluster

The *Streptomyces* sp. Cl 58-27 was subjected to genome sequencing in order to identify the secondary metabolite gene cluster responsible for the production of kendomycin E and to reveal the genetic differences that cause the modifications in relation to the kendomycin cluster from *S. violaceruber*. After sequencing, the genome was assembled into 38 contigs, with an overall size of 8,527,007 bp and a G+C content of 70.9%, which is typical for *Streptomyces*.

**Figure 2:**
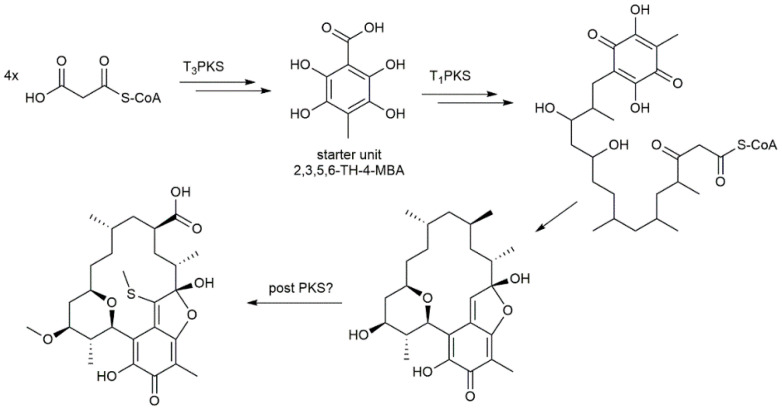
Schematic of biosynthetic pathway for the synthesis of kendomycin natural products.

The secondary metabolite biosynthesis gene cluster of *S.* sp. Cl 58-27 was examined using the bioinformatic online tool antiSMASH [15]. According to antiSMASH annotation and analysis, the genome of *S.* sp. Cl 58-27 harbors 31 gene clusters including 7 terpene; 4 PKS; 4 NRPS; others such as siderophore, bacteriocin, butyrolactone, melanine, ectoine clusters and several hybrid clusters. At first glance, no clusters seemed to fit for kendomycin production. Only detailed analysis of all biosynthetic clusters led to the discovery of genes that fit to the kendomycin biosynthesis. One cluster, annotated as an NRPS cluster, shows cluster similarity to kendomycin and was found to contain some of the genes involved in kendomycin biosynthesis. Based on MIBiG [18] analysis of those genes, we found the following similarities to known kendomycin gene clusters: a “thioesterase” gene with 53% sequence homology to a type I PKS gene (module 7–8), a hypothetical protein with 46% sequence homology to a FAD-dependent monooxygenase and a PKS gene with 52% sequence homology to type I PKS gene (module 1–3) (Table S2). Other identified biosynthetic gene clusters in antiSMASH did not show any obvious homology to literature kendomycin gene clusters. Thus, the gene sequences of *ken2*, *ken13* and *ken 16* were blasted in Geneious against the whole genome of Cl 58-27. As a result, parts of the cluster were also found in regions 13.2, 27.1 and 29.1 (Figure S8). For instance, region 13.2 (genes #1−27, Figure S8) contains a type III PKS (gene #20) that shows 77% identity with *ken2*, which is responsible for biosynthesis of the starter unit. Surrounding genes in region 13.2 have similarly fitting sequence homologies and thus belong to the group of genes that are needed to prepare the starter unit 2,3,4,6-TH-4-MBA (Figure 2). Furthermore, regions 27.1 and 29.1 contain several polyketide synthase genes that show the highest sequence similarity to sceliphrolactam biosynthetic genes (genes #28–47, Table S2). However, due to the blast results in Geneious and lack of other fitting genes, they must belong to the remaining parts of the kendomycin gene cluster. With this, the whole biosynthesis of kendomycin E in *S.* sp. Cl 58-27 can be reconstructed and conclusions drawn regarding how the new modifications evolve. The biosynthesis of kendomycin was first described by Bode et al. in 2000 [9]. By means of stable isotope feeding experiments with [1-^13^C]acetate, [2-^13^C]acetate, [1-^13^C]propionate, [1,2-^13^C_2_]acetate and L-[*methyl*-^13^C]methionine, they suggested the following biosynthetic pathway. Starting from acetate and methionine, synthesis of the starter unit is catalyzed by a chalcone synthase. The aliphatic core of kendomycin is then built up from six methylmalonyl-CoA and two malonyl-CoA molecules catalyzed by a type I PKS. As final steps and cleavage from the enzyme, several cyclization steps take place to produce kendomycin (Figure 2).

The whole biosynthetic gene cluster responsible for kendomycin biosynthesis was later published by Wenzel et al. [14]. The cluster consists of 21 genes, including the core genes *ken12*–*14* and *ken16*, which encode type I PKSs (Figure S8b). Comparing the genes identified in *S.* sp. Cl 58-27 for kendomycin biosynthesis with literature data revealed that within the type III PKS part of the kendomycin gene cluster, an enoyl-CoA-hydratase is missing but an additional O-methyltransferase is present (Table S2). Nevertheless, the aromatic starter unit appears to be the same for both kendomycin E and known kendomycins. The *O*-methyltransferase could be instead responsible for methylation of the hydroxy group at C-7 in the tetrahydropyran ring, which does not occur in the biosynthesis of other derivatives. The tetrahydropyran ring and the aliphatic chain, which is later connected to the aromatic part, are synthesized by a type I PKS. In regions 27.1, 29.1 and 31.1, six type I PKSs genes were found, containing a total of eight KS modules, as reported for the known kendomycins. However, in module 2, malonyl-CoA is used instead of methylmalonyl-CoA, resulting in the missing methyl group at position C-8. Module 5 must be skipped entirely during the biosynthesis, since one methylmalonate incorporation is missing between C-12 and C-13. In module 6, methylmalonyl-CoA is again inserted in the growing chain; however, the methyl group thereof is later carboxylated. Supposably, carboxylation is carried out by a cytochrome P450 monooxygenase. The corresponding gene was found close to the type III PKS, which synthesizes the starter unit. The gene shows 51% identity with the *ncmO* gene, which is supposed to catalyze carboxylation in nocamycin biosynthesis (Table S2) [17]. However, further deletion experiments are necessary to confirm this finding. Another peculiarity is the insertion of a thiomethyl group. We can only speculate that the thiomethyl group is derived from *N*-acetyl-L-cysteine (NAC), as reported for kendomycin D [13]. However, as in kendomycin C, the NAC group is further modified to a simple thiomethyl and it is not clear which genes catalyze those two reactions. No genes were found that could carry out the insertion of NAC and the subsequent modification. Presumably, genes from elsewhere in the genome are responsible for these reactions.

## 3. Materials and Methods

### 3.1. Cultivation, Metabolite Extraction and Dereplication

The strain *Streptomyces* sp. Cl 58-27 was precultivated in a 100 mL flask filled with 10 mL of TSB (tryptic soy broth 30 g/L) at 28 °C and 180 rpm on a rotary shaker for 24 h. For the main culture, 100 mL of DNPM medium (dextrin 40 g/L, Bacto Soytone 7.5 g/L, yeast extract 5 g/L and MOPS (4-morpholinepropanesulfonic acid) 21 g/L, pH 6.8) in a 500 mL flask was inoculated with 1 mL of preculture and cultivated for 5 days at 28 °C and 180 rpm on a rotary shaker. After cultivation, the biomass and culture liquid were extracted separately with a mixture of acetone/methanol (1:1) and ethyl acetate, respectively. The solvent was evaporated to dryness, and the residue was dissolved in 300 µL of MeOH. High-resolution LC-MS data were collected on a Dionex Ultimate 3000 UHPLC system (Thermo Fisher Scientific, Waltham, MA, USA) with an Acquity UPLC BEH C18, 100 × 2.1 mm, 1.7 µm column (Waters Corporation, Milford, MA, USA) as the stationary phase, using a linear gradient from 5% B (acetonitrile +0.1% formic acid) against A(ddH_2_O +0.1% formic acid) to 95% B and coupled to a PDA detector operating at 200–600 nm. The coupled LTQ Orbitrap mass spectrometer (Thermo Fisher Scientific, Waltham, MA, USA) was operated at *m*/*z* 200–2000. Data were analyzed with Thermo Xcalibur software version 3.0.63. Dereplication was performed by comparison of the exact masses with the Dictionary of Natural Products database version 10.0 (CRC Press, Boca Raton, FL, USA).

### 3.2. Isolation, Purification and Structure Elucidation

For the isolation and purification of the target compounds, *Streptomyces* sp. Cl 58-27 was cultivated in 5 L of DNPM (50 × 500 mL flasks with 100 mL medium) as described above. After cultivation, the culture liquid was separated from the biomass and extracted twice with ethyl acetate. The solvent was removed under reduced pressure, and the residue was dissolved in methanol and centrifuged. The pellet was discarded, and the supernatant was dried, resulting in 2.5 g of raw material. The crude extract was dissolved in 20 mL of methanol and fractionated in two portions (10 mL per run) by size exclusion chromatography using Sephadex^®^ LH 20 (Sigma Aldrich, Darmstadt, Germany) as the stationary phase (100 cm column, filled with 300 mL of Sephadex in methanol) and methanol as the eluent. Fractions were collected every 15 min with a speed of 1–2 drops per second. Every third fraction was analyzed by LC-MS on a Dionex Ultimate 3000 UPLC system using an Acquity BEH C18, 50 × 2.1 mm, 1.7 µm d_p_ column (Waters Corporation, Milford, MA, USA) and a mobile phase of ddH_2_O +0.1% formic acid (A)/acetonitrile +0.1% formic acid (B), 5–95% B over 9 min, at a flow rate of 0.6 mL/min, coupled to an amaZon SL speed mass spectrometer (Bruker, Billerica, MA, USA) with an ESI source and a mass range of *m*/*z* 200–2000. Fractions containing targeted compounds were further purified on a Waters AutoPurification™ system coupled to a single quadrupole mass detector (Waters, Milford, MA, USA). As the stationary phase, a Nucleodur C18 HTEC, 250 × 21 mm, 5 µm column (Macherey-Nagel, Düren, Germany) and water (A)/methanol (B) + 0.1% formic acid were used. A linear gradient from 5% to 95% B in 15 min was applied. The fractions containing kendomycin E were further purified by semipreparative high-performance liquid chromatography (HPLC) using the following equipment: Agilent 1100 HPLC (Agilent Technologies, Santa Clara, CA, USA) equipped with a Nucleodur C18 HTEC column (250 × 10 mm, 5 µm, Macherey-Nagel, Düren, Germany) and a DAD detector operating at 200–600 nm. A linear gradient used solvent A (MQ-H_2_O +0.1% formic acid) against solvent B (acetonitrile +0.1% formic acid) starting from 30% B and increasing to 95% B over 15 min with a flow rate of 4.5 mL/min at 45 °C.

The NMR spectra were acquired in deuterated dimethyl sulfoxide (DMSO-d_6_) and MeOD_4_ at 298 K on a Bruker Avance III 700 or 500 MHz spectrometer, both equipped with a 5 mm TXI cryoprobe. NMR shifts were relative to the residual solvent signal DMSO-d_6_ at δ 2.50 ^1^H and MeOD_4_ at δ 3.31 ^1^H, or to the solvent itself at δ 39.5 (DMSO-d_6_) and δ 49.0 (MeOD_4_) for ^13^C measurements. NMR data were analyzed using Topspin, version 3.5 pl7 (Bruker, United States) and Spectrus Processor 2018.2.3 (ACD/Labs, Toronto, Canada).

Kendomycin E (**1**). Yellow powder; 1.6 mg; UV (MeOH) λ_max_ 218, 291, 386 nm; δ_H_ (500 MHz, methanol-*d_4_*): 5.41 (d, H-5), 3.96 (t, H-9), 3.48 (m, H-7), 3.40 (s, 3xH-20), 2.81 (m, H-6), 2.78 (s, 3xH-24), 2.23 (quint, H-14), 2.09 (m, H-16), 2.00–1.34 (m, 2xH-8), 1.88 (s, 3xH-25), 1.84 (m, H-12), 1.53–1.01 (m, 2xH-11), 1.52–1.41 (m, 2xH-10), 1.35 (m, 2xH-15), 1.30–1.23 (m, 2xH-13), 1.25 (d, 3xH-23), 0.95 (d, 3xH-19), 0.89 (d, 3xH-21); δ_C_ (500 MHz, methanol-*d_4_*): 182.6 (C-3), 181.5 (C-22), 168.5 (C-1), 157.1 (C-18), 150.1 (C-4), 127.2 (C-18a), 118.3 (C-17), 110.8 (C-4a), 104.7 (C-2), 80.4 (C-7), 76.1 (C-5), 70.9 (C-9), 57.8 (C-20), 46.3 (C-14), 43.1 (C-13), 42.8 (C-16), 39.0 (C-6), 37.5 (C-8), 33.7 (C-15), 33.2 (C-11), 32.6 (C-10), 29.0 (C-12), 19.3 (C-21), 16.2 (C-24), 14.5 (C-19), 14.5 (C-23), 7.9 (C-25). HRESIMS *m*/*z* 523.2365 [M + H]^+^ (calc. for C_27_H_38_O_8_S) (meas. 523.2358 [M + H]^+^).

### 3.3. Antimicrobial Susceptibility Test

Minimum inhibitory concentrations (MICs) were determined according to standard procedures. All bacterial isolates were handled according to standard procedures or were part of our internal collection and were cultured under conditions recommended by the depositor. *S. aureus* Newman, *E. coli* (wt), *E. coli* (ΔacrB), *B. subtilis* and *P. aeruginosa* PA14 were inoculated in Mueller Hinton Broth (Sigma Aldrich) and incubated under shaking conditions for 24 h at 37 °C; *A. baumannii* and *C. freundii* at 30 °C. *C. neoformans*, *P. anomala*, *M. hiemalis* and *C. albicans* were cultivated in Myc medium under shaking conditions for 24 h at 30 °C. *M. smegmatis* was cultivated in M7H9 medium under shaking conditions at 37 °C for 48 h. *S. aureus* and *E.coli* were subcultured on CASO agar plates, *M. smegmatis* was subcultured on Middlebrook 7H10 agar plates (M7H10) with 10% oleic acid-albumin-dextrose-catalase OADC Enrichment and 5% sheep blood agar plates, respectively, and incubated for 24 h at their optimal growth temperature. The tested compounds were prepared as DMSO stocks (10 mg/mL). Single colonies of the bacterial strains were suspended in 0.9% NaCl and McFarland was adjusted to 0.5 using a densitometer. The bacterial suspension was diluted 1:100 in the corresponding broth to achieve a final inoculum of approximately 104 CFU/mL. Serial dilutions of compounds (0.06 to 128 μg/mL) were prepared in sterile 96-well plates and the bacterial suspension was added. Growth inhibition was assessed after overnight incubation (24–48 h) at 30–37 °C. MICs were determined as the lowest compound concentration where no visible growth was observed.

### 3.4. Genome Sequencing and Bioinformatics

For DNA isolation, *Streptomyces* sp. Cl 58-27 was inoculated in TSB medium and grown at 28 °C on a rotary shaker (180 rpm) for 3 days. High-quality DNA was isolated using standard procedure [19]. The purity and concentration of the isolated genomic DNA was determined using a Nanodrop 2000 spectrophotometer (Thermo Fisher Scientific). The obtained genomic DNA was sequenced using Illumina for short-read sequencing and PacBio Sequel Il/Ile, Nanopore for long-read sequencing as platforms by Novogene, UK. De novo assembly was completed using Newbler assembly v2.8 with default settings. Genome annotation was carried out using Prokka v1.11 and GenDB 2.0 platform [20,21]. Genome analysis was performed using antiSMASH (https://antismash.secondarymetabolites.org/#!/start, accessed on 5 September 2021) [15], MIBig [18] and Geneious software version 9.1.2.

## 4. Conclusions

The discovery of new antibiotics has become more challenging than ever. The frequent rediscovery of known metabolites and the successively faster development of bacterial resistance against newly discovered compounds are major hurdles. Nevertheless, the search for new compounds is indispensable, and the past has shown that even small changes in the structural features of antibiotics can have a major impact on their effectiveness. We have reported here a new derivative of the kendomycin family of natural products that differs in degree of methylation. Additionally, as a first variant, it possesses a shorter alkyl chain and a rare carboxylation of a methylmalonyl unit that occurs in-line or as a posttranslational modification. The gene cluster responsible for the production of kendomycin E in *S.* sp. Cl 58-27 was identified, which enabled us to also reveal the gene putatively involved in the rare carboxylation of the methyl group. Future experiments are needed to confirm this finding.

## Supplementary Materials

The following are available online. Table S1: NMR spectroscopic data of kendomycin E (**1**), acquired in CD_3_OD (500 MHz). Table S2: Genes in the kendomycin biosynthetic gene cluster from *Streptomyces* sp. CL 58-27 and their closest similar proteins. Figure S1: LC-HRMS chromatogram of crude extract of CL 58-27. Peak at RT 11.24 min coincides with the targeted compound kendomycin E. Figure S2: Mass chromatogram of kendomycin E showing the [M + H]^+^ peak with a mass of 523.24 Da. Figure S3: ^1^H NMR spectrum of kendomycin E measured in CD_3_OD (500 MHz). Figure S4: ^13^C NMR spectrum of kendomycin E measured in CD_3_OD (500 MHz). Figure S5: ^1^H-^1^H COSY spectrum of kendomycin E measured in CD_3_OD (500 MHz). Figure S6: HSQC spectrum of kendomycin E measured in CD_3_OD (500 MHz). Figure S7: HMBC spectrum of kendomycin E measured in CD_3_OD (500 MHz). Figure S8: (a) Kendomycin gene cluster in *Streptomyces* sp. Cl58-27. (b) Kendomycin gene cluster in *Streptomyces violaceruber* (strain 3844-33C) identified by Wenzel et al.

## Sample Availability

Samples of the compound kendomycin E are available from the authors.
