# Supplementary material for: New Kendomycin Derivative Isolated from Streptomyces sp. Cl 58-27
Source: Molecules. 2021 Nov 12;26(22):6834. doi: 10.3390/molecules26226834 (PMC8620246; doi:10.3390/molecules26226834)
Supplement: Supplementary file 1 [file molecules-26-06834-s001.zip › molecules-1342517-supplementary.pdf]

Supplementary Materials

Table S1. NMR spectroscopic data of kendomycin E (**1**), acquired in CD_3OD (500 MHz).

Position	δ_C [a]	δ_H [b]	COSY	HMBC
**1**	168.5, C	-	-	-
**2**	104.7, C	-	-	-
**3**	182.6, C	-	-	
**4**	150.1, C	-	-	-
**4a**	110.8, C	-	-	-
**5**	76.1, CH	5.41, d (10.6)	2.81-0.94-3.48	14.1-38.9-70.9-80.4-110.8-127.2-150.1-182.6
**6**	39.0, CH	2.81, m	0.94-3.48-5.41	76.1-14.1
**7**	80.4, CH	3.48, m	2.00-2.81	57.9-70.9-76.1-80.4-14.1-38.9
**8**	37.5, CH_2	2.00-1.34, m	3.48-3.96	39.0-80.4
**9**	70.9, CH	3.96, t (10.9)	2.00/1.34-1.52/1.41	32.6
**10**	32.6, CH_2	1.52-1.41, m	3.96	19.3-29.0-43.1-70.9
**11**	33.2, CH_2	1.53-1.01, m	1.84	19.3-29.0
**12**	29.0, CH	1.84, m	0.89	-
**13**	43.1, CH_2	1.30-1.23, m	-	19.3-29.0-33.2
**14**	46.3, CH	2.23, quint (8.1)	1.35	29.0-33.7-42.8-181.5
**15**	33.7, CH_2	1.35, m	-	181.5
**16**	42.8, CH	2.09, m	1.25	14.5-33.7-46.3-118.3
**17**	118.3, C	-	-	-
**18**	157.1, C	-	-	-
**18a**	127.2, C	-	-	-
**19**	14.5, CH_3	0.95, d (6.8)	2.81	38.9-76.1-80.4
**20**	57.8, CH_3	3.40, s	-	80.4
**21**	19.3, CH_3	0.89, d (6.4)	1.84	29.0-33.2-43.1
**22**	181.5, C	-		-
**23**	14.5, CH_3	1.25, d (6.7)	2.09	33.7-42.8-118.3
**24**	16.2, CH_3	2.78, s	-	127.2-157.1
**25**	7.9, CH_3	1.88, s	-	104.7-127.2-168.5-182.6

[a] followed by multiplicity; [b] followed by multiplicity and coupling constant J in Hz.

Table S2. Genes in kendomycin biosynthetic gene cluster from *Streptomyces* sp. CL 58-27 and their closest similar proteins.

Gene #	Function	Closest Similar Protein, Identity (%)
1	methyltransferase	O-methyltransferase, 45% (migrostatin, *Streptomyces platensis subsp. rosaceus*)
2	unknown	-
3	methyltransferase	methyltransferase *ken9*, 54% (kendomycin, *Streptomyces violaceoruber*)
4	unknown	-
5	cytochrome P450	Cytochrome P450 *ncmO*, 51% (nocamycin, *Saccharothrix syringae*)
6	*nmrA* family protein	Hypothetical protein, 31% (dynemicin A, *Micromonospora chersina)*

7	LuxR family transcriptional regulator	Regulatory protein, 40% (salinilactam, *Salinispora tropica* CNB-440)
8	short-chain dehydrogenase/reductase SDR	Oxidoreductase, 47% (lugdunomycin, *Streptomyces* sp. QL37)
9	transcriptional regulator	Hx1R transcriptional regulator, 51% (microansamycin, *Micromonospora* sp. HK160111)
10	unknown	-
11	FAD linked oxidase domain protein	Oxidase, 57% (microansamycin, *Micromonospora* sp. HK160111)
12–15	Binding proteins	-
16	short-chain dehydrogenase/reductase SDR	2,3-dihydroxybenzoate-2,3-dehydrogenase, 37% (carboxamycin, *Streptomyces* sp. NTK 937)
17	short-chain dehydrogenase/reductase SDR	Oxidoreductase, 45% (lugdunomycin, *Streptomyces* sp. QL37)
18	unknown	-
19	thioesterase	Thioesterase, 64% (caniferolide A, *Streptomyces caniferus)*
20	chalcone and stilbene synthase domain protein (T3PKS)	Type III PKS *ken2*, 77% (kendomycin, *Streptomyces violaceoruber)*
21	unknown	-
22	enoyl-CoA hydratase	Dioxygenase *ken4*, 61% (kendomycin, *Streptomyces violaceoruber)*
23	pyruvate oxidase/decarboxylase	Benzoyl formate decarboxylase *ken5*, 73% (kendomycin, *Streptomyces violaceoruber)*
24	aldehyde dehydrogenase	Benzaldehyde dehydrogenase *ken6*, 75% (kendomycin, *Streptomyces violaceoruber)*
25	enoyl-CoA hydratase	enoyl-CoA hydratase *ken3/7, 80%* (kendomycin, *Streptomyces violaceoruber)*
26	unknown	-
27	Beta-ketoacyl synthase	Polyketidesynthase *merC*, 75% (meridamycin, *Streptomyces violaceusniger)*
28	Short chain dehydrogenase/reductase	Polyketidesynthase *sceO*, 61% (sceliphrolactam, *Streptomyces sp. SD85)*
29	Transcriptional regulator	Transcriptional regulator *sceP*, 58% (Sceliphrolactam, *Streptomyces sp. SD85*)
30	unknown	-
31	β ketoacylsynthase (KS-AT-DH-KR/KS-AT-DH-KR/KS-AT-DH-KR)	Polyketidesynthase *SceQ*, 67% (sceliphrolactam, *Streptomyces sp. SD85*)
32	β ketoacylsynthase (KS-AT-KR)	Polyketidesynthase *sceR*, 73% (sceliphrolactam, *Streptomyces sp. SD85*)
33	β ketoacylsynthase (KS-AT-DH-KR	Polyketidesynthase *sceS*, 70% (sceliphrolactam, *Streptomyces sp. SD85*)
34	unknown	-
35	unknown	-
36	cytochrome P450	cytochrome P450 *sceD*, 80% (sceliphrolactam, *Streptomyces sp. SD85*)

37	cytochrome P450	cytochrome P450 *sceE*, 76% (sceliphrolactam, *Streptomyces sp. SD85*)
38	dehalogenase	Haloalkane dehalogenase *sceF*, 74% (sceliphrolactam, *Streptomyces sp. SD85*)
39	acyltransferase	Transacylase *SceG*, 70% (sceliphrolactam, *Streptomyces sp. SD85*)
40	Binding protein	Binding protein *sceH*, 64% (sceliphrolactam, *Streptomyces sp. SD85*)
41	AMP dependent synthetase and ligase	AMP dependent synthetase and ligase *sceI*, 73% (sceliphrolactam, *Streptomyces sp. SD85*)
42	AMP dependent synthetase and ligase	AMP dependent synthetase and ligase *sceJ*, 70% (sceliphrolactam, *Streptomyces sp. SD85*)
43	Diaminopemelate decarboxylase	Diaminopemelate decarboxylase *MacK*, 72% (sceliphrolactam, *Streptomyces sp. SD85*)
44	unknown	-
45	unknown	-
46	T1PKS (KS-AT-DH-KR, KS-AT-DH-KR)	Polyketidesynthase *sceN*, 68% (sceliphrolactam, *Streptomyces sp. SD85*)
47	T1PKS (KS-AT-DH-KR)	Polyketidesynthase *sceO*, 74% (sceliphrolactam, *Streptomyces sp. SD85*)
48	thioesterase	53% kendomycin type I PKS (module 7-8)
49	unknown	-
50	unknown	-
51	modular PKS	56% mycotrienine

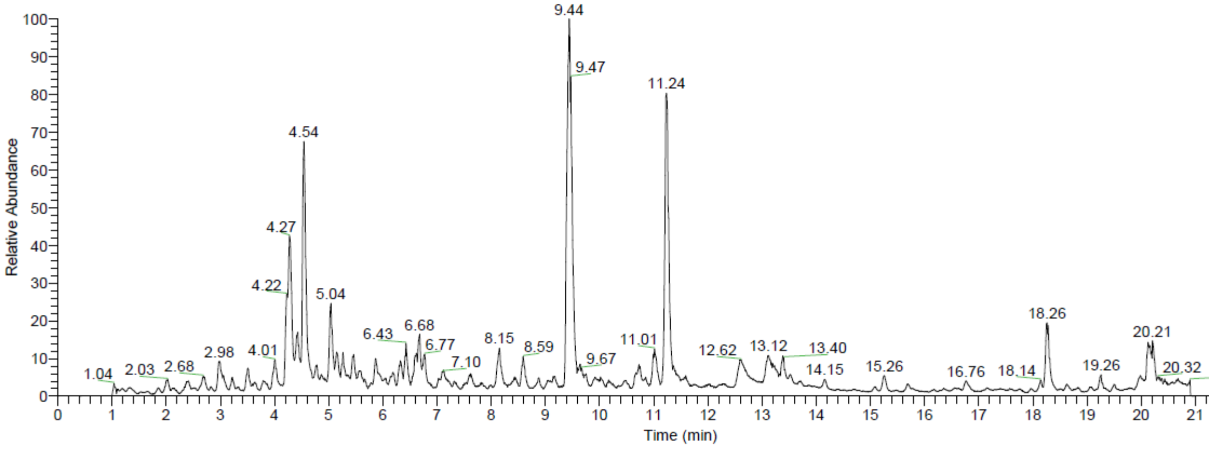

Figure S1. LC-HRMS chromatogram of crude extract of CL 58-27. Peak at RT 11.24 min coincides with the targeted compound kendomycin E.

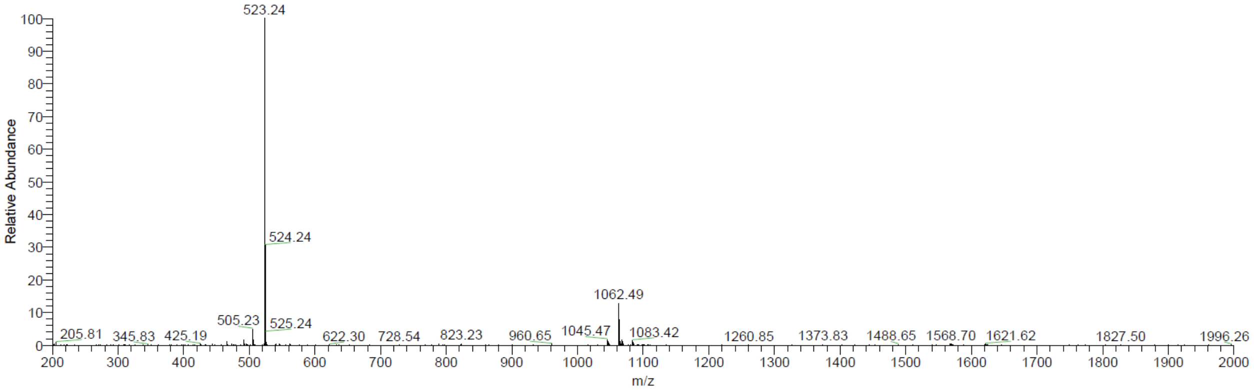

Figure S2. Mass chromatogram of kendomycin E showing the $[M+H]^+$ peak with a mass of 523.24 da.

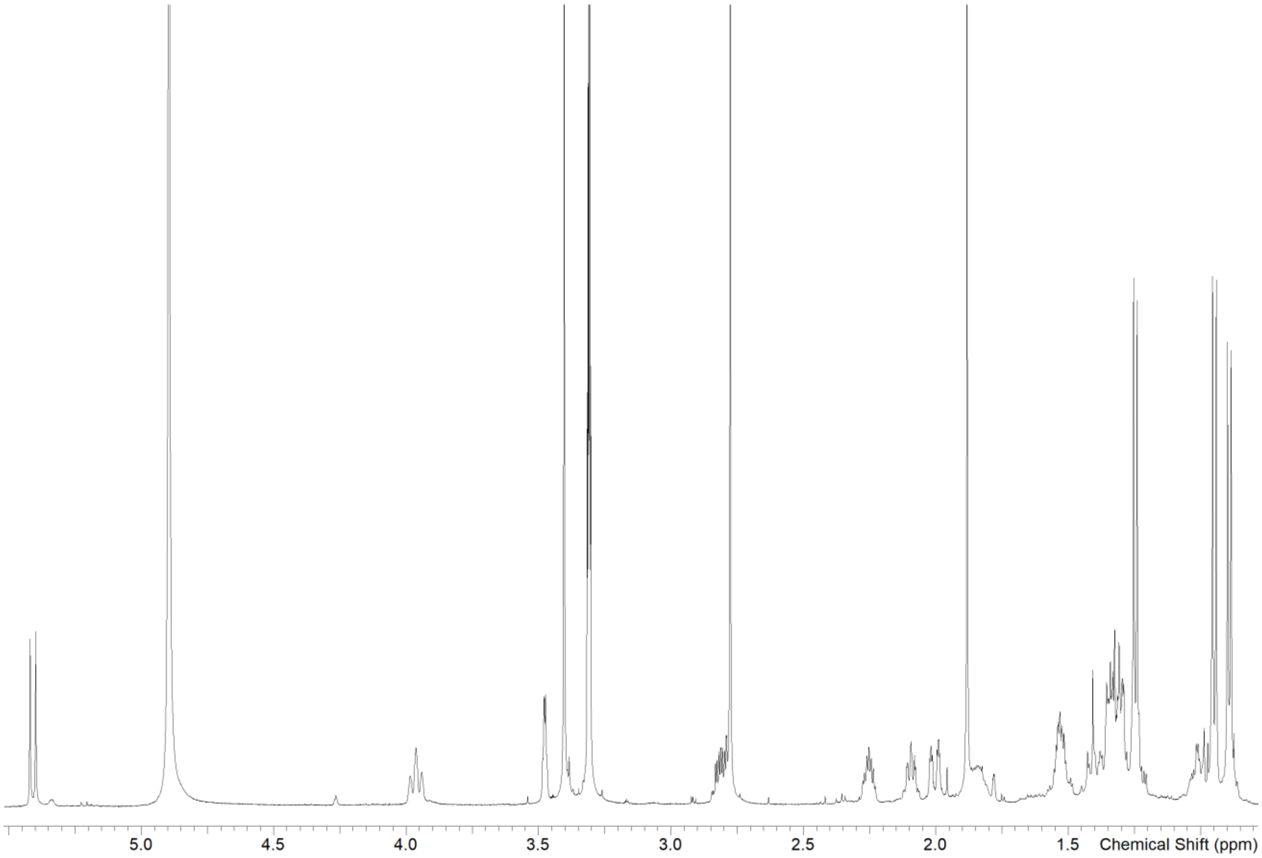

Figure S3. ^{1}H NMR spectrum of kendomycin E measured in CD_3OD (500 MHz).

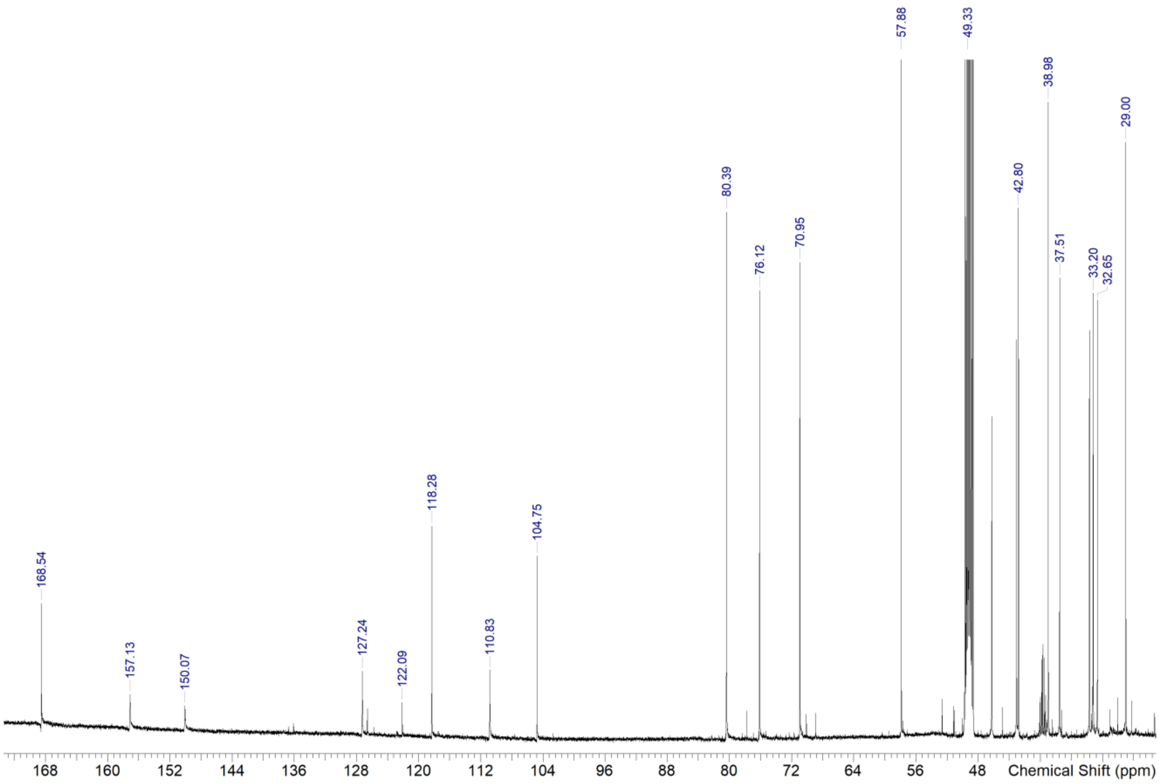

Figure S4. ^{13}C NMR spectrum of kendomycin E measured in CD_3OD (500 MHz).

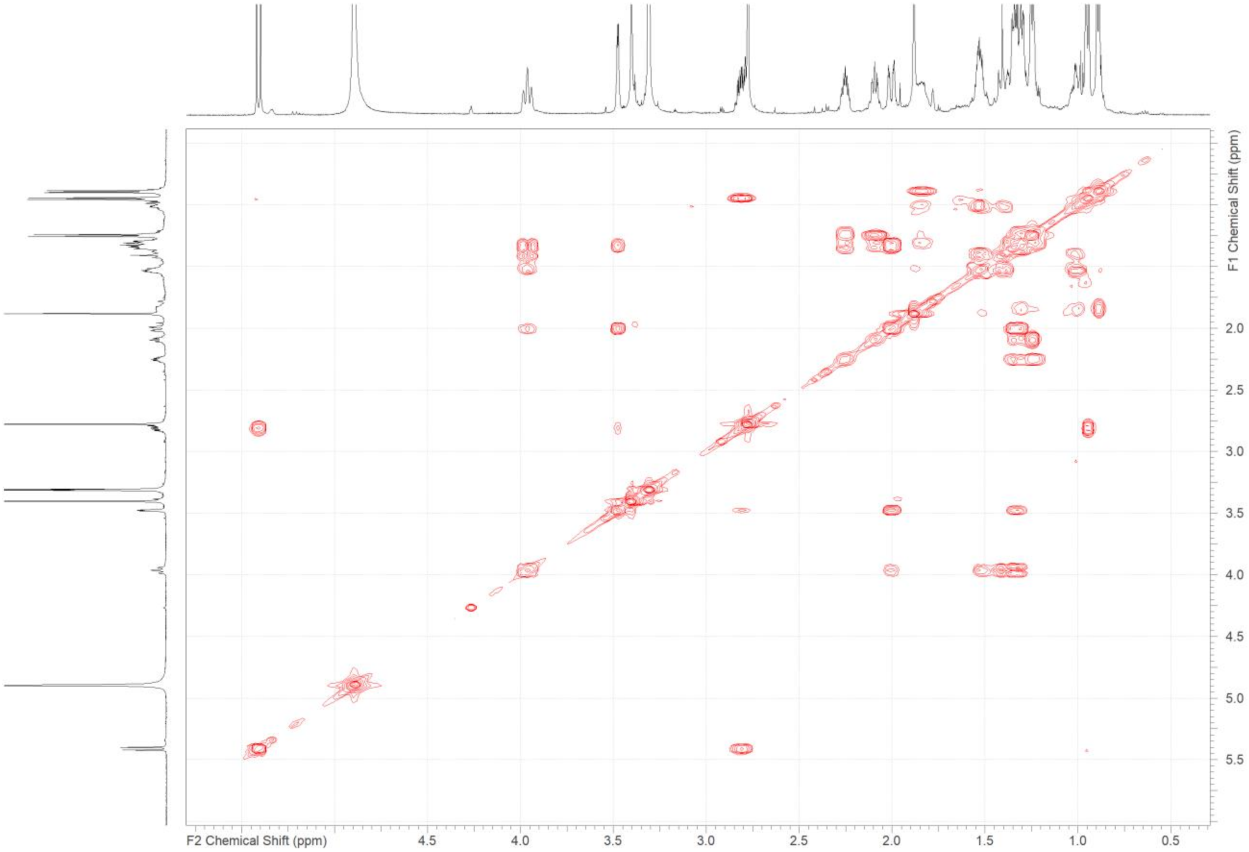

Figure S5. ^{1}H-^{1}H COSY NMR spectrum of kendomycin E measured in CD_3OD (500 MHz).

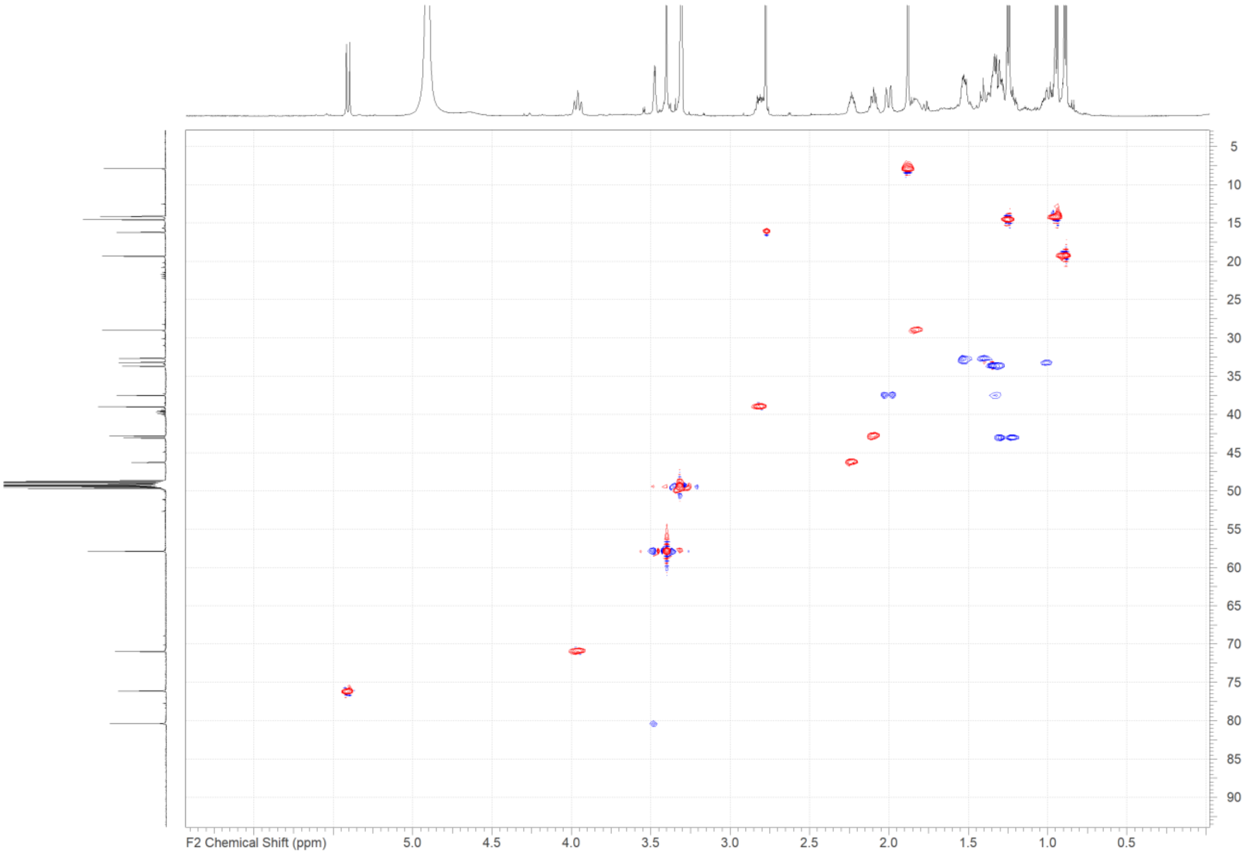

Figure S6. HSQC spectrum of kendomycin E measured in CD_3OD (500 MHz).

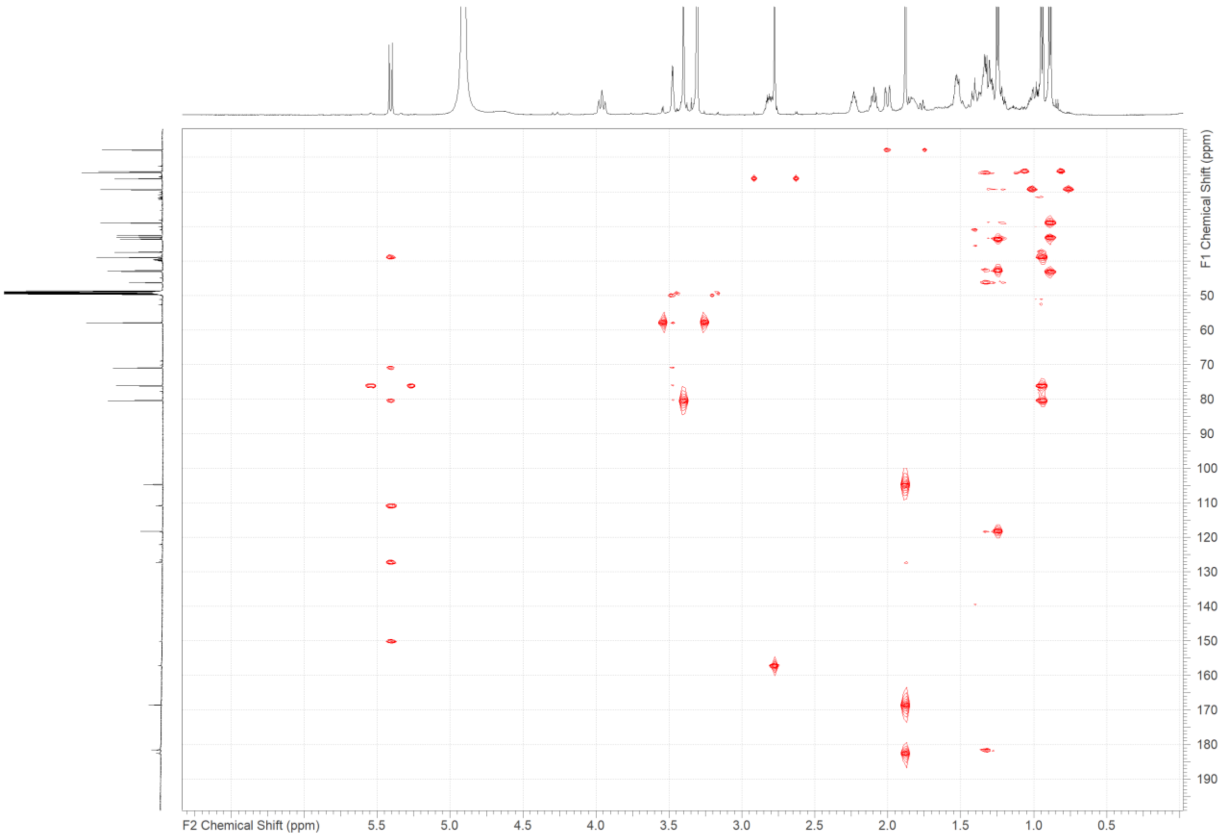

Figure S7. HMBC spectrum of kendomycin E measured in CD_3OD (500 MHz).

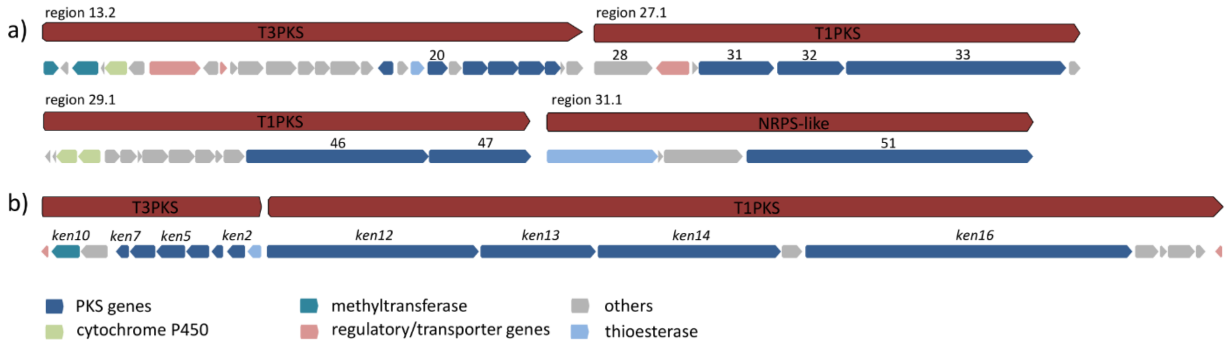

Figure S8. (**a**) Kendomycin gene cluster in Streptomyces sp. Cl58-27. (**b**) Kendomycin gene cluster in Streptomyces violaceruber (strain 3844-33C) identified by Wenzel et al. [1].

References

1. Wenzel, S.C.; Bode, H.B.; Kochems, I.; Müller, R. A type I/type III polyketide synthase hybrid biosynthetic pathway for the structurally unique ansa compound kendomycin. *Chembiochem* **2008**, *9*, 2711–2721, doi:10.1002/cbic.200800456.
